# Evaluation of PRVC and SIMV ventilation techniques on hemodynamic metrics and arterial blood gases in ICU patients with multiple trauma: A randomized, triple-blind study

**DOI:** 10.2478/jccm-2025-0043

**Published:** 2025-10-31

**Authors:** Majid Vatankhah Tarbebar, Saeed Kashani, Fatemeh Darsareh, Tayyebeh Zarei, Bibi Mona Razavi, Latifeh Farzanfar, Mehrdad Sayadinia, Pourya Adibi, Mansour Shabani, Mehrdad Malekshoar, Milad Mohammadi

**Affiliations:** Anesthesiology, Critical Care and Pain Management Research Center, Hormozgan University of Medical Sciences, Bandar Abbas, Iran; Student Research Committee, Faculty of Nursing and Midwifery, Hormozgan University of Medical Sciences, Bandar Abbas, Iran

**Keywords:** mechanical ventilation, SIMV, PRVC, multiple traumas, intensive care

## Abstract

**Background:**

In the Intensive Care Unit (ICU), mechanical ventilation is frequently employed to assist critically injured patients with breathing. The two conventional methods are SIMV and PRVC. This research sought to evaluate these techniques, particularly concerning patient stability and the preservation of optimal blood gas levels.

**Methods:**

We carried out a parallel-group, randomized, triple-blind clinical trial. One hundred two patients with multiple traumas admitted to the ICU were randomly allocated to either the SIMV group or the PRVC mode group. The main outcome was measured through blood hemodynamic parameters, blood pressure, and heart rate in mechanically ventilated patients with multiple traumas. The secondary outcome measured was the composition of arterial blood gases (pH, PaCO2, PaO2, HCO3, and SpO2).

**Result:**

The average age in the SIMV and PRVC groups was 38.53±16.29 and 38.04±15.26 years, respectively, showing no statistical significance. Arterial blood gas parameters, including arterial blood pH, PaCO2, PaO2, HCO3, and SpO2, were similar in the SIMV and PRVC groups at the beginning of admission and 8 and 12 hours after admission, and there was no significant difference. Comparing vital signs including blood pressure (systolic, diastolic, and mean arterial pressure) and heart rate was similar in the SIMV and PRVC groups at the beginning of admission and 8 and 12 hours after admission.

**Conclusion:**

No significant justification was identified to favor one approach over the other for trauma patients receiving ventilatory support. Both groups stayed consistent regarding vital signs and other health indicators.

## Introduction

For patients experiencing multiple traumas, mechanical ventilation in the intensive care unit is essential. Many factors rely on the selection of a ventilation method for clinical results. Possible benefits include improved oxygen delivery, respiratory regulation, cardiovascular steadiness, and optimized gas exchange whenever feasible [1].

Synchronized Intermittent Mandatory Ventilation (SIMV) is a key mode of ventilation utilized in respiratory therapy to deliver crucial mechanical ventilation assistance. In this mode, regulated breaths are blended and aligned with the patient’s spontaneous breaths [2]. SIMV was first created in the 1970s as a technique to assist patients who rely on mechanical ventilation in their weaning process. SIMV became well-known and was the most commonly utilized ventilatory mode for weaning, with 90.2% of hospitals choosing SIMV in a survey carried out in the 1980s [3].

Ventilator modes are advancing in complexity, and settings that once needed clinician input and adjustments are now being automated. Pressure Regulated Volume Control (PRVC) serves as an instance of an adaptive targeting method, as it involves the adaptive adjustment of inspiratory pressure to achieve the intended minute ventilation/tidal volume [4]. PRVC modifies pressure and tidal volume to meet the patient’s requirements. This mode establishes a particular tidal volume by adjusting inspiratory pressure according to lung mechanics and the patient’s capacity [5].

To evaluate the efficiency of mechanical ventilation, it is essential to measure circulatory parameters and examine arterial blood gases. This is particularly crucial for individuals with debilitating conditions such as multiple episodes of trauma [6]. This information allows experts to determine the effectiveness and safety of various ventilation methods. By closely tracking these indicators, physicians can adjust their ventilation techniques to meet unique patient requirements and help avert fatalities.

The objective of this study is to compare the effects of PRVC and SIMV ventilation modes on hemodynamic parameters and arterial blood gas levels in patients with multiple traumas. We aimed to investigate if this method would affect gas exchange and enhance patient outcomes, which would subsequently benefit care for this essential group.

## Material and method

We carried out a parallel-group, randomized, triple-blind clinical trial. The Ethics Committees for Scientific Research approved the ethics code IR.HUMS.REC.1401.176, and the protocol has been registered under ID: (IRCT20240929063203N1). The patient’s guardians signed the written informed consent form. Due to the urgent situation of multiple trauma needing swift mechanical ventilation, we established a deferred consent protocol sanctioned by the Institutional Ethics Committee, which is ethically sound and recognized in emergency care studies. Patients who met the criteria were included within the initial 2 hours of ICU admission, and research personnel reached out to families within 6–12 hours to thoroughly explain the study protocol, potential risks, benefits, and the option to withdraw, utilizing translated standard information sheets.

The study’s inclusion criteria consisted of patients aged 16 to 70 years who were experiencing multiple traumas and required mechanical ventilation in the ICU. This study focused on hemodynamically stable multiple trauma patients with non-pulmonary injuries who required mechanical ventilation primarily for airway protection and management of altered consciousness, rather than respiratory failure. The criteria for excluding participants from the study included any deaths occurring within 24 hours after admission to the intensive care unit, significant previous histories of pulmonary diseases, instability in hemodynamic parameters and arterial blood gases, as well as cases of lung contusion and acute respiratory distress. Additionally, patients with multiple trauma who had lung injuries, pre-existing airway disease, required emergency surgery, needed CPR, and those deemed hemodynamically unstable were not included.

One hundred two patients with multiple traumas admitted to the ICU were randomly allocated to either the SIMV group or the PRVC mode group. Randomization was conducted using sealed envelopes that held an odd number for the SIMV ventilation method group, while the even numbers were assigned to the PRVC ventilation method group. Subsequently, the necessary figures for the specified Number were produced for the consumer. Each Number was printed on a card, placed in a bag, and sealed while the patient number was noted on each bag. Envelope number 1 was given to the initial patient involved in the study; envelope number 2 was provided to the second patient, and this continued accordingly. To prevent this intentional manipulation, the individual who created the envelopes was not the same person who recruited patients and distributed the envelopes. To remove physician bias in allocating treatment among patients, a non-physician registered patients regarding each group and the registration of patients for each category. In conclusion, the patients were unconscious and did not know which method they were under. Secondly, the observer was unaware of the allocation of patients to each group, and thirdly, the statistical analyst was unaware of the allocation of individuals to groups.

### Intervention

Eligible patients were divided into two groups. Group A (n=51) received SIMV ventilation method (tidal volume: 6 cc/kg, respiratory rate: 12–14, FiO2: 40–50%, pressure support: 8–12 mmH2O, PEEP: 5 cmH2O, I/E ratio: 1:2, and Trigger sensitivity: 3 L/min). Group B (n= 51) received PRVC ventilation method (tidal volume: 6 cc/kg, respiratory rate: 12–14, FiO2: 40–50%, pressure support: 8–12 mmH2O, PEEP: 5 cmH2O, I/E ratio: 1:2, and Trigger sensitivity: 3 L/min). Ventilation for both groups was carried out with the Respina device, and sedation was provided with two medications: midazolam at 1–2 mg/h and fentanyl at 50–100 mg/h.

During the final stages of the treatment period, patients were systematically transitioned off the ventilator using Pressure Support Ventilation (PSV) mode that provided pressure support. This procedure involved stepwise reductions of the support pressure while evaluating the patient’s ability to breathe spontaneously prior to tube extubation.

### Indications for Mechanical Ventilation:

Patients were intubated and mechanically ventilated for the following indications:

– Airway protection due to decreased level of consciousness (GCS ≤ 8)
– Prevention of aspiration in unconscious trauma patients
– Facilitation of deep sedation for trauma management
– Anticipated prolonged need for sedation and monitoring

Patients with primary respiratory failure or acute lung injury were excluded from the study.

### Monitoring

The patients were monitored through different parameters. Supine position with arm at heart level was selected for all patients.

Hemodynamic parameters:

– Blood Pressure: Measured using a calibrated automatic oscillometric device every 8 hours
– Systolic BP: Normal range 90–140 mmHg
– Diastolic BP: Normal range 60–90 mmHg
– Mean Arterial Pressure (MAP): Normal range 70–100 mmHg
– Heart Rate: Monitored using continuous ECG monitoring every 8 hours.
– Normal range: 60–100 beats per minute
– Arterial Blood Gas Analysis: Performed by 2mL arterial blood drawn under sterile conditions, using a calibrated blood gas analyzer every 12 hours
– PH: Normal range 7.35–7.45
– PaCO2: Normal range 35–45 mmHg
– PaO2: Normal range 80–100 mmHg
– HCO3: Normal range 22–26 mEq/L
– SpO2: Normal range 92–100%
– Complications Assessment: Pneumothorax and pneumomediastinum evaluated by daily chest X-ray
– Clinical examination: Documented using a standardized assessment form every 24 hours.
– Quality Control Measures: All measurements were performed by trained ICU nurses. Equipment calibration is performed daily. Standard operating procedures were followed for all measurements. Data recorded in standardized forms.

ICU and anesthesiology specialists adjust parameters based on the patient’s ABG within 24 hours if any imbalance exists.

### Study outcomes

The main outcome was measured through blood hemodynamic parameters, blood pressure (systolic, diastolic, and mean arterial pressure), and heart rate in mechanically ventilated patients with multiple traumas.

#### Primary Outcome

The primary outcome of this study was defined as the difference in PaO2 levels between SIMV and PRVC ventilation modes in patients with multiple trauma, as PaO2 is a critical indicator of oxygenation efficacy in mechanically ventilated patients.

### Power Analysis

We aimed to achieve a statistical power of 80% (β = 0.20) to detect clinically meaningful differences between the two ventilation modes, with a type I error rate of 5% (α = 0.05).

#### Sample Size Calculation

The sample size calculation was based on data from a previous study by El-Rahman Ali et al. [7], where:

– SIMV group: PaO2 standard deviation = 3.64 mmHg
– PRVC group: PaO2 standard deviation = 9.50 mmHg
– Minimum clinically meaningful difference (δ) = 4 mmHg

Using the formula for comparing two independent means:

$$\text{n}={\left({\text{Z}}_{1}-\alpha {/}_{2}+{\text{Z}}_{1}-\beta \right)}^{2}\times \left({\sigma_{1}}^{2}+{\sigma_{2}}^{2}\right)/\delta^{2}$$

Where:

Z_1_−α/_2_: 1.96 (for α = 0.05)
Z_1_−β: 0.84 (for β = 0.20, power = 80%)
σ_1_: 3.64 (standard deviation for SIMV group)
σ_2_: 9.50 (standard deviation for PRVC group)
δ: 4 (minimum clinically meaningful difference)

**Calculation:** n = (1.96 + 0.84)^2^ × (3.64^2^ + 9.50^2^) / 4^2^ n = (2.80)^2^ × (13.25 + 90.25) / 16 n = 7.84 × 103.50 / 16 n = 50.70 ≈ 51 patients per group

#### Actual Power of the Study

With the achieved sample size of 51 patients per group (total n = 102), and considering the actual standard deviations observed in our study:

– SIMV group: PaO2 standard deviation = 28.43 mmHg (at 12h)
– PRVC group: PaO2 standard deviation = 25.93 mmHg (at 12h)

The actual power of our study to detect a 4 mmHg difference in PaO2 between groups was calculated to be approximately 85%, which exceeds our target power of 80%, indicating adequate statistical power for detecting clinically meaningful differences in the primary outcome.

### Statistical Analysis

In this research, descriptive data analysis was conducted by computing mean, standard deviation, and percentage. When comparisons were required, comparative techniques like the independent T-test and Chi-square test were employed with a significance threshold of 0.05. All computations were carried out using SPSS software version 22.

## Results

Out of the 125 patients screened, 23 patients (18.4%) were excluded due to issues related to consent, comprising 8 patients due to family unavailability (6.4%), 12 patients due to family refusal to take part (9.6%), and 3 patients due to delays in obtaining consent (2.4%).

The average age in the SIMV and PRVC groups was 38.53±16.29 and 38.04±15.26 years, respectively, showing no statistical significance (p=0.876). Arterial blood gas parameters, including arterial blood pH, PaCO2, PaO2, HCO3, and SpO2, were examined in patients of the study groups. These parameters were similar in the SIMV and PRVC groups at the beginning of admission and 8 and 12 hours after admission, and there was no significant difference (Table 1).

**Fig. 1.**
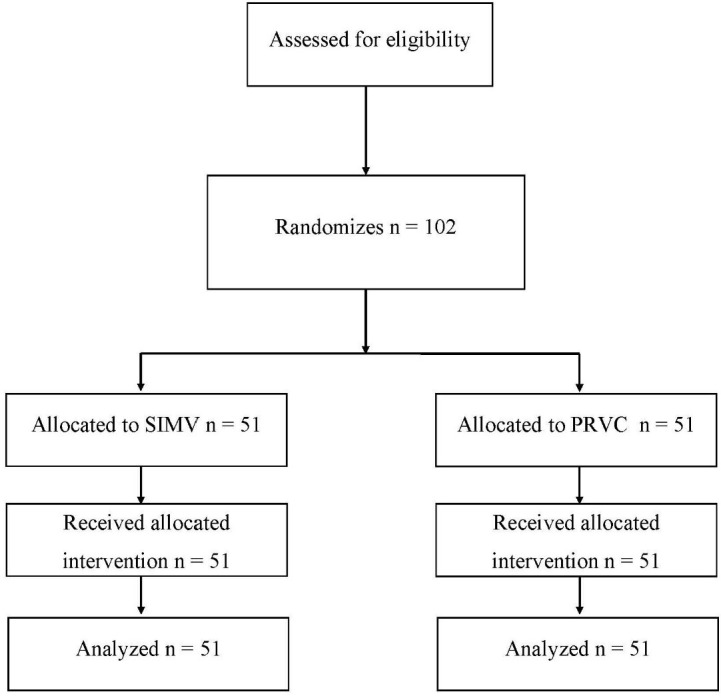
Consort flowchart

**Table 1.** Arterial blood gases in Patients of the studied groups

**Variable /Hospitalization hours (h)**	**Group SIMV (51=n)**	**Group PRVC (51=n)**	**P**
Arterial blood pH			
0h	7.40±0.06	7.38±0.07	0.166
12h	7.38±3.04	7.39±0.07	0.313

PaCO2			
0h	38.47±6.48	39.91±7.28	0.293
12h	40.85±16.10	38.45±6.14	0.324

PaO2			
0h	98.35±26.81	97.68±26.08	0.898
12h	95.22±28.43	100.91±25.93	0.229

HCO3			
0h	24.26±3.09	23.72±2.89	0.363
12h	25.67±12.16	24.99±8.55	0.746

SpO2			
0h	99.10±1.28	98.88±2.08	0.529
8h	98.86±1.72	98.78±1.93	0.829
16h	98.29±2.59	98.84±2.02	0.236

Data were presented as mean±SD. P<0.05 is statistically significant. SD: Standard deviation

Comparing vital signs, such as systolic blood pressure, diastolic blood pressure, mean arterial pressure, and heart rate, patients of the study groups showed similar results in the SIMV and PRVC groups at the beginning of admission and 8 and 12 hours after admission, and there was no significant difference (Table 2).

**Table 2.** Heart Rate, Systolic Blood Pressure, and Diastolic Blood Pressure in the Studied Groups

**Variable / Hospitalization hours (h)**	**Group SIMV (51=n)**	**Group PRVC (51=n)**	**P**
Heart Rate			
0h	86.41±14.11	88.88±14.80	0.390
8h	87.04±14.68	88.47±14.49	0.621
12h	88.20±13.83	86.67±13.46	0.573

Systolic Blood Pressure			
0h	121.79±15.40	121.57±19.54	0.964
8h	121.16±15.39	122.14±17.47	0.764
12h	124.49±15.93	133.86±87.91	0.455

Diastolic Blood Pressure			
0h	50.61±36.59	37.10±37.96	0.070
8h	49.76±35.05	44.80±36.59	0.486
12h	50.20±35.41	39.75±35.04	0.137

Mean Arterial Pressure			
0h	74.34±24.93	65.26±26.13	0.075
8h	73.56±23.53	70.58±25.15	0.537
12h	74.96±23.87	71.12±58.88	0.669

Data were presented as mean±SD. <0.05 is statistically significant. SD: Standard deviation

Patients in the study groups were also evaluated for the development of pneumothorax and pneumomediastinum. None of the patients in the study groups experienced these complications.

## Discussion

Numerous studies have been conducted in recent years aimed at maximizing benefits and reducing risks from mechanical ventilation in this group of patients since the key factor for the clinician early in mechanical ventilation is selecting an appropriate mode for the patient [ 7, 8, 9].

This study examined SIMV and PRVC ventilation methods involving 102 patients with multiple traumas (51 in each group). Our results indicate that at both admission and post-admission times of eight and twelve hours, the arterial blood gas parameters (SpO2, HCO3, PaO2, PaCO2, pH) did not exhibit significant differences among the groups. Likewise, vital signs (systolic/diastolic blood pressure, mean arterial pressure, and heart rate) showed no differences at those times, nor did the two groups. Generally speaking, both SIMV and PRVC modes have demonstrated comparable outcomes in terms of ABG values and hemodynamic parameters for ICU patients with multiple injuries. Several studies have been carried out on the benefits of PRVC mode in comparison to other mechanical ventilation modes [ 5,6,10,11].

In a study conducted by Aghadavoudi et al., 106 patients with brain injuries in the ICU were randomly allocated to receive either PRVC or SIMV modes. The results of this study showed that in SIMV mode, the Rapid Shallow Breathing Index (RSBI) is notably higher than in PRVC at the 8-hour mark. The pattern of RSBI was notably distinct as well. Additionally, a notable variation was observed in the trend of the PaO2/FiO2 ratio. It was, therefore, determined that PRVC exhibited superior hemodynamic stability and ventilation outcomes when compared to SIMV in trauma patients [11]. Lastly, these two studies do not agree, which could arise from individual variability amongst participants or can be due to single-center study design.

A recent study by Wakeel et al. investigated VCV versus SIMV in 100 patients with traumatic brain injuries. They reported VCV produced better respiratory results than SIMV, with a lower ventilatory rate (18.3 vs. 19.5 breaths/min, p=0.03), better PaO_2_ levels (90.2 vs. 85.6 mmHg, p=0.02), and improved CO_2_ clearance (PaCO_2_ 37.0 vs. 39.1 mmHg, p=0.01). VCV also demonstrated better trends in neurological recovery (improvement in GCS score 48% vs. 34%)[12].

These results differ from the study results, in which SIMV and PRVC yielded similar results in a cohort of polytrauma patients. The reason for this difference could be variability in patient characteristics and underlying disease processes. Patients with brain injuries tend to have more elaborate neuro-respiratory interactions compared to our study, which concentrated on hemodynamically stable trauma patients who needed mechanical ventilation for airway management instead of respiratory support. Furthermore, their study’s comparison of VCV and SIMV differs from our focus on PRVC versus SIMV.

Another study examined PRVC and SIMV breathing modes on 80 patients suffering from acute respiratory failure as a result of severe liver disease showed notable variations in the PO2/FiO2 ratio along with static and dynamic compliance, supporting PRVC for improved oxygenation and decreased duration of mechanical ventilation [13].

The discrepancies observed between the earlier studies and our results may stem from variations in selection methods for the populations examined, as well as significantly differing sample sizes in both studies. More studies are needed to make a better conclusion.

The strength of our study is that in this research, both SpO2 and SaO2 metrics were utilized to enhance the precision and thoroughness of evaluating patients’ oxygenation levels. Utilizing both parameters concurrently facilitates data comparison and validation, and if a notable disparity exists between them (potentially caused by various elements like hemoglobinopathy or technical issues), it enables deeper identification and examination. This combined method enhances the dependability of the findings and minimizes measurement inaccuracies [14,15,16]. The research encountered several limitations. One of these constraints was that the sample size was limited. For example, a small sample size might hinder the ability to generalize these results to other trauma patients with acute injuries in various regions and countries. The other limitation is that this research is a single-center trial. Ultimately, an additional limitation of this study might be that the period of observation was possibly too brief to detect long-term effects or changes in outcomes as time progressed. The results of this study have not been modified for certain confounders, including the level of severity and comorbid conditions.

Due to the strong expertise of the medical personnel and doctors at this center, SIMV and PRVC ventilation modes have been employed for numerous trauma patients; nonetheless, we think that alternative modes like pressure-controlled or assisted ventilation could yield comparable results, as seen in BIPAP, for example. In this research, we did not utilize the respiratory index P(A-a). While the alveolar-arterial oxygen gradient serves as a key measure of gas exchange efficiency in the lung, this study opted to concentrate on standard and readily available parameters like PaO2, PaCO2, and SaO2, which can be directly determined from arterial blood gas analysis. All these limitations should be considered in further trials.

## Conclusion

In summary, this research revealed no notable differences in arterial blood gas parameters, vital signs, or complications between the groups of multiple trauma patients receiving either SIMV or PRVC ventilation modes. These results stand in stark contrast to other research that demonstrated certain advantages of PRVC in specific patient populations. Both approaches seem to be equally effective for patients with multiple traumas; nevertheless, we must carry out additional multi-center studies with larger sample sizes to confirm these findings and investigate possible long-term outcomes. Further studies should investigate the efficacy of these ventilation modes in various subgroups of critically ill patients to enhance ventilation strategies and better patient outcomes for those in need of intensive care units.
